# Autologous adipose tissue injection versus platelet-rich plasma (PRP) injection in the treatment of knee osteoarthritis: a randomized, controlled study – study protocol

**DOI:** 10.1186/s12891-020-03345-8

**Published:** 2020-05-20

**Authors:** Paweł Bąkowski, Jakub Kaszyński, Joanna Wałecka, Kinga Ciemniewska-Gorzela, Kamilla Bąkowska-Żywicka, Tomasz Piontek

**Affiliations:** 1 Department of Orthopedic Surgery, Rehasport Clinic, Poznan, Poland; 2 Institute of Bioorganic Chemistry Polish Academy of Sciences, Poznan, Poland; 3 Department of Spine Disorders and Pediatric Orthopedics, University of Medical Sciences Poznan, Poznan, Poland

**Keywords:** Autologous subcutaneous adipose tissue, Platelet rich plasma, Knee OA, Intra- articular injection

## Abstract

**Background:**

Knee osteoarthritis (OA) is a common, chronic, progressive and degenerative disease which affects patients’ quality of life and may cause disability and social isolation. OA is a huge economic burden for the patient and a large strain for the whole healthcare system. Articular cartilage has a small potential to repair, with progressively more clinicians emphasizing cellular therapy. Subcutaneous fat tissue in human body is a large reservoir of mesenchymal stem cells (MSCs) and is been harvested in minimally invasive, simple procedure. Up to date there is no prospective randomized controlled studies demonstrating effectiveness and role of adipose tissue injections in OA treatment. The purpose of this study is to assess functional and clinical changes among patients with symptomatic knee OA treated with intra-articular injections of autologous adipose tissue or platelet rich plasma (PRP) and to compare efficacy of both therapeutic methods.

**Methods:**

This is a prospective, randomized, controlled study. Patients who meet inclusion criteria will be allocated to Fat Tissue group or PRP group randomly. Subjects will receive an intra articular injection with autologous adipose tissue and PRP respectively. Patients will be assessed five times: before treatment and 1, 3, 6 and 12 months after the treatment. The assessment consists of patient reported outcome measures (The Knee injury and Osteoarthritis Outcome Score, International Knee Documentation Committee 2000, the Western Ontario and McMaster Universities Osteoarthritis Index, the Health Questionnaire EQ- 5D- 5 L), three functional tests (The Timed Up and Go Test, The 5 Times Sit to Stand Test, The 10 m Walk Test) and Maximal Isometric Voluntary Contraction.

**Discussion:**

This study protocol has several strengths and weaknesses. One of strongest point of this study is the wide, multidimensional functional assessment which will give a large amount of objective data. On the other hand, lack of blinding has to be considered as a risk of both subject and investigator bias.

**Trial registration:**

name of registry: ClinicalTrials.gov, trial registration number: NCT04321629, retrospectively registered on date of registration.

## Background

Knee osteoarthritis (OA) is a common, chronic, progressive and degenerative disease which causes irreversible structural changes, like cartilage loss, subchondral bone sclerosis and osteophyte formation. Those changes lead to clinical symptoms, such as joint stiffness, loss of function, crepitus, effusions and pain. This disease affects patients’ quality of life, and may cause disability and social isolation [1, 2]. Two types of OA exist, primary or idiopathic and secondary. Primary does not have a clear origin in contrast to the secondary, which is caused by known medical condition or trauma [3].

OA is a huge economic burden for the patient and a large strain for whole healthcare system [4, 5]. There are several treatment options for knee OA [1–3, 6, 7]. Guidelines have been published by organizations such as European League Against Rheumatism (EULAR) or the National Institute for Health and Clinical Excellence (NICE) [5]. The choice of an adequate method depends on given patients’ joint condition and symptoms intensity. Patient education, weight loss, aerobic and strength training are first line recommendations. Next stages include paracetamol, topical and oral non-steroidal anti-inflammatory drugs (NSAIDs) and a variety of options for symptom relief, like manual therapy, transcutaneous electrical nerve stimulation and other physical therapy methods. In some cases, when conservative treatment fail, more invasive treatment like arthroscopy, partial or total arthroplasty is needed [5].

Articular cartilage has a limited potential to repair, with progressively more clinicians emphasizing cellular therapy [8]. Platelet rich plasma (PRP) is a well-proven method in OA treatment [9–12]. Promising results have been found in OA treatment with Bone-Marrow Derived Stem Cells (BMSCs) [13], but it has been noticed that adipose tissue may be a better source of Mesenchymal Stem Cells (MSCs) [14]. Concentration level of MSCs is much higher in adipose tissue than in bone marrow (2% vs 0.02% respectively) [1]. Moreover, subcutaneous tissue in human body is a large reservoir of MSCs and is been harvested in minimally invasive, simple procedure.

Autologous subcutaneous fat tissue obtained by a lipoaspiration process is widely available at clinics, despite limited evidences of its effectiveness. Up to date there is no prospective randomized controlled studies demonstrating effectiveness and role of adipose tissue injections in OA treatment.

## Design

This is a prospective, randomized, controlled study. The purpose of this study is to assess functional and clinical changes among patients with symptomatic knee OA treated with intra-articular injections of autologous adipose tissue or PRP and to compare the efficacy of both therapeutic methods. We hypothesize that adipose tissue injections will improve patients’ quality of life and functional status and will decrease pain level significantly more than PRP injections. In addition to the functional tests and muscle strength measurement, the patient reported outcome measures (PROMs) of the knee joint function and quality of life will be used to assess each participant.

The study design meets CONSORT, MIBO and SPIRIT guidelines.

### Inclusion and exclusion criteria

**Fig. 1:**
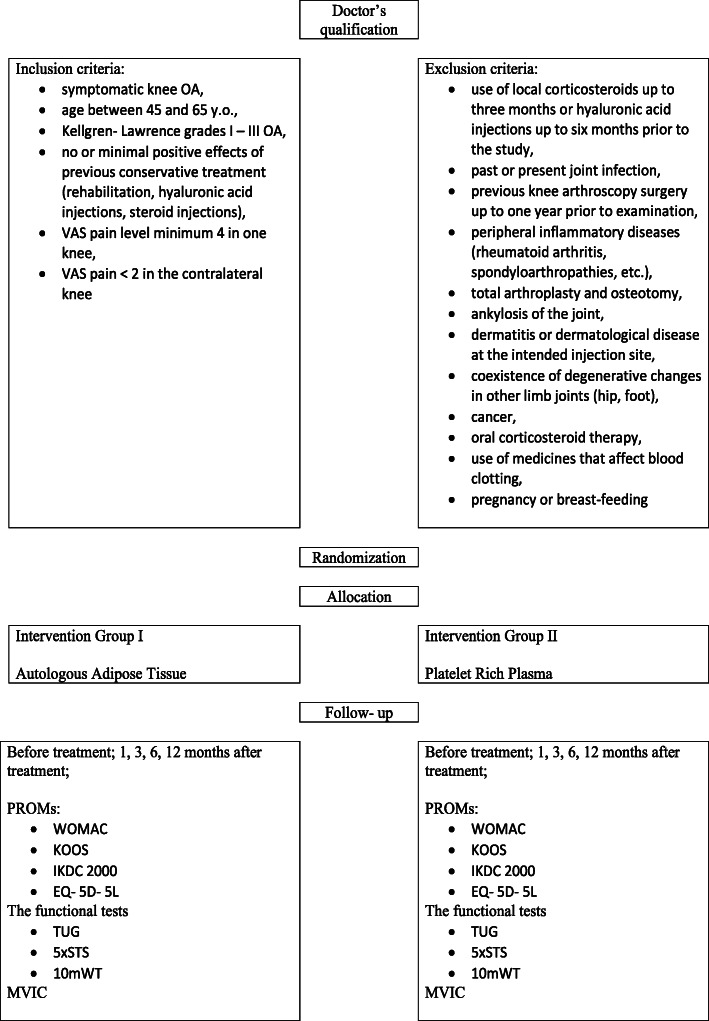
Study design

All activities related with the study will be performed at the Department of Spine Disorders and Pediatric Orthopedic Surgery, Poznan University of Medical Sciences and Rehasport Clinic in Poznan, Poland. These include patient identification, explanation of all procedures, treatment and functional assessment. The same inclusion criteria have been established for an Experimental Group (subjects treated with autologous fat tissue) and a Control Group (subjects treated with PRP). Those criteria consist of: symptomatic knee OA, age between 45 and 65 y.o., Kellgren- Lawrence grades I – III OA, no or minimal positive effects of previous conservative treatment (rehabilitation, hyaluronic acid injections, steroid injections), VAS pain level minimum 4 in one knee, VAS pain < 2 in the contralateral knee. Those patients who meet inclusion criteria will be allocated to Fat Tissue Group or PRP Group randomly (Fig. 1). The exclusion criteria were as follows: use of local corticosteroids up to 3 months or hyaluronic acid injections up to 6 months prior to the study, past or present joint infection, previous knee arthroscopy surgery up to 1 year prior to examination, peripheral inflammatory diseases (rheumatoid arthritis, spondyloarthropathies, etc.), total arthroplasty and osteotomy, ankylosis of the joint, dermatitis or dermatological disease at the intended injection site, coexistence of degenerative changes in other limb joints (hip, foot), cancer, oral corticosteroid therapy, use of medicines that affect blood clotting, pregnancy or breast-feeding. Before randomization, patients will sign a suitable consent form. All participants will be obliged not to use any anti-inflammatory drugs for the entire duration of the study. Moreover, every volunteer will receive a brochure approved by Research and Development Department which contains all of the information regarding protocol (procedures, outcomes assessments, schedule of visits, researchers team, possible complications). Draw is made at the Doctor’s room after subject’s qualification and the participant choose the number of the group 1 or 2, Fat Tissue Treatment or PRP treatment, respectively. At the stage of treatment there is no possibility for patients to be blinded because the procedure requires adipose tissue harvesting. However, during all other stages, the procedure will be blinded, in a database each participant will have a special code to minimize the risk of identification as well as reporter biases.

### Lipoaspiration procedure

Lipoaspiration will take place in the operating room under general anesthesia for patient’s and doctor’s comfort. The most frequent donor site is abdomen [14, 15]. The patient will be placed in a supine position. At first, two small incisions at the level of umbilicus will be made by the trained orthopaedic surgeon (TP or PB). Then Klein solution (saline with lidocaine and epinephrine) will be infused to reduce bleeding. Ten minutes is required for infiltration. Next step is a liposuction performed with a thin cannula inserted through incisions. Finally, skin sutures and the pressure dressing will be applied. To minimize risk of bleeding and hematoma, an elastic belt will be recommended, as well as partial weight bearing within first 2 weeks. Harvested adipose tissue will be prepared in a Lipogems kit [14]. The final product will be transferred into 10-ml syringes. About 10 ml of the product will be injected into the affected knee joint. After several hours of observation, the patient will leave the clinic with 2 elbow crutches. For the first 2 weeks, partial weight-bearing will be allowed as tolerated. After 2 weeks, full-weight loading will be allowed. Patients will be instructed to perform physical exercises, including cycling, swimming and full-body exercises. No physical therapy will be recommended.

### PRP procedure

PRP preparation takes place in an outpatient clinic. The whole process, from blood collection to PRP injection, takes approximately 10 min. It takes places in the treatment room at room temperature and day light exposure. No commercial kit are used. 10-ml sterile collecting tubes containing citrate will be placed in a centrifuge (Centrifuge MPW- 223e) with a tilting rotor. Rotation will last 7 min at 2320 * g. After centrifugation, PRP will be collected up to 3 ml for separate 10-ml syringe. 3 ml of PRP will be injected into the knee joint. Procedure will be repeated three times in 7 day interval.

### Injection procedure

Joint injection will be performed by TP or PB in the same manner for both groups: patient placed in supine position, affected knee extended, a 21-Gauge needle inserted into the suprapatellar pouch, in case of joint effusion – aspiration of synovial fluid and finally administration of autologous fat tissue or PRP.

For the first 2 weeks after injections patients will be asked to limit their activity. 2 weeks after the third injection, normal activity will be allowed. Patients will be instructed to perform physical exercises, including cycling, swimming and full-body exercises. No physical therapy will be recommended.

### Outcomes measurement

Patients will be assessed five times: before treatment and 1, 3, 6 and 12 months after the treatment (Table 1). The PROMs consist of the four questionnaires: The Knee injury and Osteoarthritis Outcome Score (KOOS) [16–18], International Knee Documentation Commitee 2000 (IKDC 2000) [19, 20], the Western Ontario and McMaster Universities Osteoarthritis Index (WOMAC) [21, 22], the Health Questionnaire EQ- 5D- 5 L [23, 24].

**Table 1:** The study procedures schedule

Visit	1	2	3	4	5
Month (m)	0	1 m	3 m	6 m	12 m
Study qualification	X				
Consent form signing	X				
Autologous fat tissue/ PRP intra- articular injection	X				
Questionnaires:					
KOOS	X	X	X	X	X
IKDC 2000	X	X	X	X	X
WOMAC	X	X	X	X	X
EQ-5D-5 L	X	X	X	X	X
Functional tests:					
TUG	X	X	X	X	X
5xSTS	X	X	X	X	X
10mWT	X	X	X	X	X
Strength measurement:					
MVIC	X	X	X	X	X

Moreover, three functional tests will be performed to assess patient’s functional status: The Timed Up and Go Test (TUG) [25], The 5 Times Sit to Stand Test (5xSTS) [26], The 10 m Walk Test (10mWT) [27]. To assess strength parameters of the knee flexors and extensors the Maximal Voluntary Isometric Contraction (MVIC) will be measured. Each test will be supervised by the same one physiotherapist to avoid any interexaminer bias and discrepancies during testing.

### PROMs

The Knee injury and Osteoarthritis Outcome Score is currently culturally adapted in 39 languages, including polish [18, 28]. The polish version has already been validated [17]. It is used for knee OA assessment and for the evaluation of the effect of many orthopedic surgeries as well [16].

International Knee Documentation Committee 2000 has been developed to assess function, symptoms and sports activity in patients with variety of knee joint conditions such as: OA, meniscal and/or ligamentous injuries, patellofemoral pain [19]. This questionnaire also has been adapted to the polish language [20].

The Western Ontario and McMaster Universities Osteoarthritis Index has been developed for knee and/or hip joints OA symptoms assessment. This questionnaire has been extensively tested for reliability and validity in measuring changes in patients’ symptoms in affected joints [21, 22]. There is no polish cultural adaptation of WOMAC that is why WOMAC index will be calculated from KOOS [29].

The Health questionnaire EQ- 5D- 5 L measures general health status including function, physical symptoms and emotional dimensions which are relevant even for healthy individuals. EQ- 5D- 5 L is 5- level, more sensitive version of the original 3- level version (EQ- 5D- 3 L) [24]. Polish population norms for the EQ- 5D- 5 L has already been established [23].

### The functional tests

#### The timed up and go test

The Patient sits on a standard chair (45 cm height) and is asked to stand up and walk straight on in a comfortable pace to the line (placed on the floor 3 m from the chair), turn around and go back to the starting position. The result of this test is time measured with stopwatch. Time limit for this test is 3 min 30 s. If the patients fails, the reason should be recorded. The participant is allowed to use the crutches or a walker if needed [25].

#### The 5 times sit to stand test

The Patient sits on a standard chair (45 cm height) without back support with arms crossed on a chest and is asked to stand up (fully extend hips and knees) then sit down again and repeat it five times. The participant starts on the rater’s command and the rater counts loudly each repetition. The result of this test is time measured with stopwatch. The stopwatch is stopped when the subject sits down after the fifth repetition. The patient is not allowed to bounce from the chair and use any other support [26].

#### The 10 m walk test

The patient stand in upright position and is asked to walk straight on, as fast as he/she can to the line marked on the floor 10 m from the starting point. The participant starts on the rater’s command. The result of this test is time measured with stopwatch. The patient is not allowed to run but may use the crutches or a walker if needed [27].

### Maximal voluntary isometric contraction measurement

MVIC measurement is performed with Forcemeter FB 500 (AXIS, Gdansk, Poland). The patient sits on a bench with the belt around waist and legs placed freely beyond the table. The measuring belt is placed parallel to the floor just above the ankle joint with knee flexed to 90 degrees. The measuring belt length is strictly specified: for MVIC of extensors 160 cm, for MVIC of flexors 60 cm.

The procedure starts with the measuring belt pretension, then the patient is asked to extend/flex the knee as hard as he/she can and hold it for 6 s. The force is measured in Newtons [N]. The results is divided by the patient’s weight [kg] for data analysis.

### Ethics, data management and statistical analysis

All procedures used for this study have been approved by Bioethics Committee, Poznan University of Medical Sciences on 16th December 2019 (no. 868/18). The tile of the project accepted by Bioethics Committee: “Prospective functional evaluation of knee osteoarthritis treatment with autologous fragmented adipose tissue and platelet-rich plasma (PRP) – comparison of two different treatment methods”. All patients will be obliged to give written agreement for the participation in the study. Collected data will be stored at the clinic in a database created particularly for this study containing appropriate security features. In database each participant will have a special code to minimize the risk of identification of a given patient. Only patients who attended all of visits, performed each functional test and MVIC and fulfilled each questionnaire will be included in the analysis. The consequence of the absence on a visit will be exclusion from the study. Basic analysis will include descriptive statistics of demographic characteristics to assess both groups homogeneity. Such analysis will be done several times during testing to detect any confounders which may influence the balance between treatment groups. At the start we consider BMI, age, sex and knee OA grade as a possible confounders, but in a further analysis we will also look for any correlations between them and treatment effectiveness. At every time point the results will be compared between treatment and control group, what is main purpose of this study. The results obtained in each questionnaire, functional test and MVIC will create the effect across all time points. A *p*- value of < 0.05 will be considered statistically significant.

### Platelet rich plasma and adipose derived Mesenchymal stem cells in knee OA treatment- overview

Nowadays, PRP is commonly used in medicine to enhance expression of growth factors and cell rebuilding process not only in orthopedics but dermatology and dentistry as well [30, 31]. In OA treatment PRP is thought to have an influence on the whole joint environment by increasing chondrocyte proliferation [32], reduction of the inflammatory process [33, 34] and enhancing the secretion of hyaluronic acid (HA) as well [35]. It is well established that PRP is more effective in knee OA treatment than placebo (saline intra-articular injections) [12, 36–39]. More discrepancies among researchers exist when it comes to comparing the effectiveness of PRP and HA treatment. Some authors have found no differences between the groups treated with PRP and HA [34, 36], others [40–42] prove PRP injections alone or in combination with HA to be more effective than HA injections alone.

The autologous fat tissue, which contains numbers of pericytes [37, 38], serves as a kind of drugstore after the injection into the affected joint. It is thought that they secrete bioactive molecules and stimulate other type of cells by cell- cell contact [37]. The effects of autologous fat tissue activity are apoptosis inhibition, enhancement of angiogenesis and stabilization of the new vessels, stimulation of the progenitors to appropriate differentiation [37, 39]. Some authors combine intra-articular injections of autologous fat tissue with arthroscopic debridement [15, 43, 44] or microfractures [45–47], but autologous fat tissue alone may also improve degenerated joint condition [42, 48–51].

## Discussion

This study protocol has several strengths and weaknesses. Undoubtedly one of strongest point of this study is wide, multidimensional functional assessment, which will give a large amount of objective data. To our knowledge, this research is the first one which includes the battery of functional tests and MVIC as an outcome measurement tool. This study will use 4 questionnaires, including WOMAC, which is described by physicians as a gold standard for assessing the effectiveness of knee OA treatment [45, 52].

On the other hand this study has several limitations. Primarily - the lack of blinding and we consider this as a risk of both, subject and investigator bias. Next and equally important limitation is a small size of the study. Furthermore, autologous fat tissue procedure is definitely more invasive and more stressful for the patients than PRP procedure. Hence, taking all into consideration, to adopt autologous fat tissue as a knee OA therapy, based on the future results, we have to detect definite, statistically significant and clinically noticeable difference.

Our PRP procedure gives us the possibility to examine the patient and assess the reaction to intra- articular injection 3 times (3 injections) in 7 day interval. These visits are often associated with physical therapy, which consists of manual therapy and individualized exercise program. Autologous fat tissue procedure does not give us such a possibility. The doctor and physiotherapist see the patient on the day of the surgery and 2 weeks after, during a control visit, which is also associated with physical therapy. Thus, there are some discrepancies between the two procedures at the beginning of the treatment process.

It has been proven that intra- articular injection of autologous fat tissue or PRP is a safety treatment option of knee OA [46, 47]. The most common complications after the intra- articular injection are pain and swelling of treated knee, but this improve after cold compression and NSAIDs. Also there were no cancer incidents reported after autologous fat tissue or PRP implantation [47].

Subject recruitment has started after we received Bioethical Committee approval.

## Data Availability

The datasets used during the current study are available from the corresponding author on reasonable request.

## Abbreviations

OA: Knee osteoarthritis
MSCs: Mesenchymal stem cells
PRP: Platelet rich plasma
EULAR: European league against rheumatism
NICE: National institute for health and clinical excellence
NSAIDs: Non-steroidal anti-inflammatory drugs
BMSCs: Bone-marrow derived stem cells
PROMs: Patient reported outcome measures
KOOS: The knee injury and osteoarthritis outcome score
IKDC 2000: International knee documentation commitee 2000
WOMAC: Western ontario and mcmaster universities osteoarthritis index
EQ- 5D- 5 L: Health questionnaire
TUG: The timed up and go test
5xSTS: The 5 times sit to stand test
10mWT: The 10 m walk test
MVIC: Maximal voluntary isometric contraction
HA: Hyaluronic acid
